# Supplementary material for: Ralstonia solanacearum elicitor RipX Induces Defense Reaction by Suppressing the Mitochondrial atpA Gene in Host Plant
Source: Int J Mol Sci. 2020 Mar 15;21(6):2000. doi: 10.3390/ijms21062000 (PMC7139787; doi:10.3390/ijms21062000)
Supplement: Supplementary file 1 [file ijms-21-02000-s001.zip › Tables S1-S3.docx]

Table S1. ATPA homologs found from *Nicotiana benthamiana* genome database

| **Subject ID** | **Identity%** | **Aligment** | **e-value** | **Score** | **Description** |
| --- | --- | --- | --- | --- | --- |
| Niben101Scf04529g00005.1 | 58.04 | 296/510 | 0.0 | 585 | ATP synthase subunit alpha IPR005294 (ATPase, F1 complex, alpha subunit), Length = 507 |
| [Niben101Scf00167g04011.1](https://solgenomics.net/tools/blast/show_match_seq.pl?blast_db_id=267;id=Niben101Scf00167g04011.1;hilite_coords=5-493) | 59.16 | 297/502 | 0.0 | 583 | ATP synthase subunit alpha IPR005294 (ATPase, F1 complex, alpha subunit), Length = 507 |
| [Niben101Scf00854g11048.1](https://solgenomics.net/tools/blast/show_match_seq.pl?blast_db_id=267;id=Niben101Scf00854g11048.1;hilite_coords=1-320) | 89.38 | 286/320 | 0.0 | 576 | ATP synthase subunit alpha IPR005294 (ATPase, F1 complex, alpha subunit), Length = 320 |
| [Niben101Scf01816g00009.1](https://solgenomics.net/tools/blast/show_match_seq.pl?blast_db_id=267;id=Niben101Scf01816g00009.1;hilite_coords=1-282) | 98.58 | 278/282 | 0.0 | 563 | ATP synthase subunit alpha IPR005294 (ATPase, F1 complex, alpha subunit), Length = 286 |
| [Niben101Scf06849g00011.1](https://solgenomics.net/tools/blast/show_match_seq.pl?blast_db_id=267;id=Niben101Scf06849g00011.1;hilite_coords=1-243) | 98.35 | 239/243 | e-170 | 485 | ATP synthase subunit alpha IPR000194 (ATPase, F1/V1/A1 complex, alpha/beta subunit, nucleotide-binding domain), Length = 279 |
| [Niben101Scf06221g03002.1](https://solgenomics.net/tools/blast/show_match_seq.pl?blast_db_id=267;id=Niben101Scf06221g03002.1;hilite_coords=1-238) | 93.70 | 223/238 | e-157 | 454 | ATP synthase subunit alpha IPR000194 (ATPase, F1/V1/A1 complex, alpha/beta subunit, nucleotide-binding domain), Length = 278 |
| [Niben101Scf00854g07028.1](https://solgenomics.net/tools/blast/show_match_seq.pl?blast_db_id=267;id=Niben101Scf00854g07028.1;hilite_coords=44-279) | 90.68 | 214/236 | e-149 | 432 | ATP synthase subunit alpha IPR000194 (ATPase, F1/V1/A1 complex, alpha/beta subunit, nucleotide-binding domain), Length = 279 |
| [Niben101Scf02074g02068.1](https://solgenomics.net/tools/blast/show_match_seq.pl?blast_db_id=267;id=Niben101Scf02074g02068.1;hilite_coords=1-274) | 69.04 | 223/323 | e-142 | 415 | ATP synthase subunit alpha IPR000194 (ATPase, F1/V1/A1 complex, alpha/beta subunit, nucleotide-binding domain), Length = 274 |
| [Niben101Scf02074g06075.1](https://solgenomics.net/tools/blast/show_match_seq.pl?blast_db_id=267;id=Niben101Scf02074g06075.1;hilite_coords=3-230) | 67.79 | 202/298 | e-127 | 375 | ATP synthase subunit alpha IPR000194 (ATPase, F1/V1/A1 complex, alpha/beta subunit, nucleotide-binding domain), Length = 230 |
| [Niben101Scf00291g02025.1](https://solgenomics.net/tools/blast/show_match_seq.pl?blast_db_id=267;id=Niben101Scf00291g02025.1;hilite_coords=1-188) | 93.09 | 175/188 | e-120 | 355 | ATP synthase subunit alpha IPR000194 (ATPase, F1/V1/A1 complex, alpha/beta subunit, nucleotide-binding domain), Length = 188 |
| [Niben101Scf04451g00022.1](https://solgenomics.net/tools/blast/show_match_seq.pl?blast_db_id=267;id=Niben101Scf04451g00022.1;hilite_coords=5-209) | 74.18 | 181/244 | e-111 | 335 | ATP synthase subunit alpha IPR000194 (ATPase, F1/V1/A1 complex, alpha/beta subunit, nucleotide-binding domain), Length = 282 |
| [Niben101Scf09846g02018.1](https://solgenomics.net/tools/blast/show_match_seq.pl?blast_db_id=267;id=Niben101Scf09846g02018.1;hilite_coords=19-199) | 88.95 | 161/181 | e-105 | 323 | ATP synthase subunit alpha IPR000194 (ATPase, F1/V1/A1 complex, alpha/beta subunit, nucleotide-binding domain), Length = 392 |
| [Niben101Scf01510g00022.1](https://solgenomics.net/tools/blast/show_match_seq.pl?blast_db_id=267;id=Niben101Scf01510g00022.1;hilite_coords=1-171) | 90.70 | 156/172 | e-104 | 313 | ATP synthase subunit alpha IPR000194 (ATPase, F1/V1/A1 complex, alpha/beta subunit, nucleotide-binding domain), Length = 177 |
| [Niben101Scf02847g00017.1](https://solgenomics.net/tools/blast/show_match_seq.pl?blast_db_id=267;id=Niben101Scf02847g00017.1;hilite_coords=14-215) | 78.67 | 166/211 | e-103 | 313 | ATP synthase subunit alpha IPR000194 (ATPase, F1/V1/A1 complex, alpha/beta subunit, nucleotide-binding domain), Length = 215 |
| [Niben101Scf01210g00056.1](https://solgenomics.net/tools/blast/show_match_seq.pl?blast_db_id=267;id=Niben101Scf01210g00056.1;hilite_coords=1-172) | 77.56 | 159/205 | e-101 | 306 | ATP synthase subunit alpha IPR000194 (ATPase, F1/V1/A1 complex, alpha/beta subunit, nucleotide-binding domain), Length = 211 |
| [Niben101Scf09696g00036.1](https://solgenomics.net/tools/blast/show_match_seq.pl?blast_db_id=267;id=Niben101Scf09696g00036.1;hilite_coords=1-198) | 77.27 | 153/198 | e-100 | 304 | ATP synthase subunit alpha IPR000194 (ATPase, F1/V1/A1 complex, alpha/beta subunit, nucleotide-binding domain), Length = 198 |
| [Niben101Ctg12954g00001.1](https://solgenomics.net/tools/blast/show_match_seq.pl?blast_db_id=267;id=Niben101Ctg12954g00001.1;hilite_coords=1-160) | 85.09 | 137/161 | 3e-90 | 277 | ATP synthase subunit alpha IPR000194 (ATPase, F1/V1/A1 complex, alpha/beta subunit, nucleotide-binding domain), Length = 170 |
| [Niben101Scf03227g03022.1](https://solgenomics.net/tools/blast/show_match_seq.pl?blast_db_id=267;id=Niben101Scf03227g03022.1;hilite_coords=6-160) | 84.52 | 131/155 | 5e-86 | 266 | ATP synthase subunit alpha IPR000194 (ATPase, F1/V1/A1 complex, alpha/beta subunit, nucleotide-binding domain), Length = 170 |
| [Niben101Scf02929g06122.1](https://solgenomics.net/tools/blast/show_match_seq.pl?blast_db_id=267;id=Niben101Scf02929g06122.1;hilite_coords=26-207) | 73.63 | 134/182 | 8e-81 | 254 | ATP synthase subunit alpha IPR000194 (ATPase, F1/V1/A1 complex, alpha/beta subunit, nucleotide-binding domain), Length = 208 |
| [Niben101Scf03227g03023.1](https://solgenomics.net/tools/blast/show_match_seq.pl?blast_db_id=267;id=Niben101Scf03227g03023.1;hilite_coords=1-135) | 87.41 | 118/135 | 5e-75 | 237 | ATP synthase subunit alpha IPR000194 (ATPase, F1/V1/A1 complex, alpha/beta subunit, nucleotide-binding domain), Length = 143 |

Table S2. Bacterial strains and plasmids used in this study

| **Strains or plasmids** | **Relevant characteristics** | **Resources** |
| --- | --- | --- |
| **Strains** | | |
| *Ralstonia solanacearum* | | |
| FJ1003 | A wild type *Ralstonia solanacearum* strain isolated from *Nicotiana tobaccum* | Zhang *et al*., 2017 |
| *Escherichia coli* | | |
| DH5α | *F^-^ recA hsdR17 (rk^−^, mk^+^) ϕ80lacZ∆M15* | Clontech |
| Yeast | | |
| AH109 | MATa, *trp1-901, leu2-3, 112, ura3-52, His3-200, gal4, gal80, LYS2::GAL1UAS-GAL1TATA-His3* | Clontech |
| *Agrobacterium tumefaciens* | | |
| GV3101 | Rif^r^, with Ti plasmid pMP90 | Koncz and Schell, 1996 |
| **Plasmids** | | |
| pHB | Km^r^, a binary vector to express gene under control of a double CaMV 35S promoter | Mao *et al*., 2005 |
| pHB:PopA1 | Km^r^, the 1035-bp full length *PopA1* gene cloned in pHB at *Hin*dIII and *Xba*I sites | This study |
| pHB:PopA3 | Km^r^, the 750-bp DNA fragment coding for *PopA3* cloned in pHB at *Hin*dIII and *Xba*I sites | This study |
| pET41a (+) | Km^r^, IPTG-inducible expression vector | Novagen |
| pET41:PopA1 | Km^r^, a 1035-bp *PopA1* gene cloned in pET41a(+) at *Eco*RI and *Xho*I sites for expressing GST-PopA1 fusion | This study |
| pET41:PopA3 | Km^r^, a 750-bp *PopA3* gene in pET41a(+) at *Eco*RI and *Hin*dIII sites for expressing GST-PopA3 fusion | This study |
| pGBKT7 | Km^r^, GAL4(1–147) DNA-BD, TRP1, c-Myc epitope tag | Clontech |
| pGBKT7:PopA1 | Km^r^, a 1032-bp coding sequence of *PopA1* cloned in pGBKT7 at *Nde*I and *Eco*RI sites | This study |
| pGADT7 | Km^r^, SV40 NLS GAL4 AD LEU2, HAN epitope tag | Clontech |
| pGADT7:ATPA | Km^r^, a 1530-bp *atpA* gene cloned into pGADT7 at *Nde*I and *Eco*RI sites | This study |
| pCAMBIA-NLuc | Km^r^, N-terminal luciferase gene in pCAMBIA1300 vector | Chen *et al*., 2008 |
| ATPA-nLUC | Km^r^, a 1530-bp coding sequence of full length *atpA* gene cloned into pCAMBIA1300-nLUC | This study |
| pCAMBIA-CLuc | Km^r^, C-terminal luciferase gene in pCAMBIA1300 vector | Chen *et al*., 2008 |
| cLUC-PopA1 | Km^r^, a 1032-bp full length *PopA1* with the deletion of translation start codon ATG cloned into pCAMBIA1300-cLUC | This study |
| TRV1 | Km^r^, a VIGS vector encoding the replication, movement and cysteine-rich protein protoeins of tobacco rattle virus | Liu *et al.*, 2002 |
| TRV2 | Km^r^, a VIGS vector harboring the coat protein and two non-structural proteins of tobacco rattle virus | Liu *et al*., 2002 |
| TRV:*PDS* | Km^r^, a 369-bp DNA fragment of *NbPDS* cloned in TRV2 | Liu *et al*., 2002 |
| TRV:*gfp* | Km^r^, a 358-bp fragment of *gfp* gene cloned in TRV2 | Lab store |
| TRV:*atpA* | Km^r^, a 309-bp gene cloned in TRV2 at *Bam*HI and *Xba*I sites | This study |
| pCAMBIA1381 | Km^r^, plant expression vector with fusible *gusA* | CAMBIA |
| pCAMBIA:P*atpA* | Km^r^, a 1488-bp promoter sequence of *atpA* cloned into pCAMBIA1381 at *Bam*HI and *Pst*I sites | This study |
| pGDR | Km^r^, RFP transient expression vector | Goodin *et al*., 2002 |
| pGDR:PopA1 | Km^r^, a 1035-bp coding sequence of *PopA1* cloned into pGDR at *Xho*I and *Hin*dIII sites | This study |
| pGDGm | Km^r^, GFP transient expression vector | Sun *et al*., 2018 |
| pGDGm:ATPA | Km^r^, a 1530-bp *atpA* gene cloned into pGDGm at *Xho*I and *Sal*I sites | This study |
| pGDY | Km^r^, GFP transient expression vector | Goodin *et al*., 2002 |
| pGDY:ATPA | Km^r^, a 1530-bp *atpA* gene cloned into pGDY at *Xho*I and *Hin*dIII sites | This study |
| MT-rk-CD3-991 | Km^r^, a mitchrondria targteting marker construct | Nelson *et al*., 2007 |
| 1301-YC | Km^r^, derivated from pCAMBIA1301 which encodes the C-terminal portion of YFP | Li *et al*., 2011 |
| YC-ATPA | Km^r^, a 1530-bp *atpA* gene cloned in 1301-YC at *Xba*I and *Kpn*I sites | This study |
| 1301-YN | Km^r^, derivated from pCAMBIA1301 which encodes the N-terminal portion of YFP | Li *et al*., 2011 |
| YN-PopA1 | Km^r^, the 1032-bp coding sequence of full length *PopA1* cloned in 1301-YN at *Xba*I and *Kpn*I sites | This study |

**References**

Goodin, M.M.; Dietzgen, R.G.; Schichnes, D.; Ruzin, S.; Jackson, A.O. pGD vectors: versatile tools for the expression of green and red fluorescent protein fusions in agroinfiltrated plant leaves. *Plant J.* **2002**, *31,* 375–383.

Chen, H.; Zou, Y.; Shang, Y.; Lin, H.; Wang, Y., Cai, R.; Tang, X.; Zhou, J.M. Firefly luciferase complementation imaging assay for protein-protein interactions in plants. *Plant Physiol.* **2008**, *146*, 368–376.

Koncz, C.; Schell, J. The promoter of TL-DNA gene 5 controls the tissuespecific expression of chimeric genes carried by a novel type of *Agrobacterium* binary vector. *Mol. Gen. Genet.* **1986**, *204*, 383–396.

Li, Y.R.; Che, Y.Z.; Zou, H.S.; Cui, Y.P.; Guo, W.; Zou, L.F.; Biddle, E.M.; Yang, C.H.; Chen, G.Y. Hpa2 required by HrpF to translocate *Xanthomonas oryzae* transcriptional activator-like effectors into rice for pathogenicity. *Appl. Environ. Microbiol*. **2011**, *77***,** 3809-3818.

Liu, Y.; Schiff, M.; Dinesh-Kumar, S.P. Tobacco *Rar1*, *EDS1* and *NPR1/NIM1* like genes are required for N-mediated resistance to tobacco mosaic virus. *Plant J.* **2002**, *30*, 415-429.

Mao, J.; Zhang, Y.C.; Sang, Y.; Li, Q.; Yang, H.Q. A role for *Arabidopsis* cryptochromes and COP1 in the regulation of stomatal opening. Proc. Natl. Acad. Sci. USA **2005**, *102*, 12270–12275.

Sun, Q.; Li, Y.Y.; Wang, Y.; Zhao, H.H.; Zhao, T.Y.; Zhang, Z.Y.; Li, D.W.; Yu, J.L.; Wang, X.B.; Zhang, Y.L., Han, C.G. Brassica yellows virus P0 protein impairs the antiviral activity of NbRAF2 in *Nicotiana benthamiana*. *J. Exp. Bot.* **2018**, *69*, 3127-3139.

Zhang, C.; Chen, H.; Cai, T.; Deng, Y.; Zhuang, R.; Zhang, N.; Zeng, Y.; Zheng, Y.; Tang, R.; Pan, R.; Zhuang, W. Overexpression of a novel peanut NBS-LRR gene *AhRRS5* enhances disease resistance to *Ralstonia solanacearum* in tobacco. *Plant Biotech. J.* 2017, *15*, 39-55.

Table S3. Primers used for molecular cloning and qRT-PCR in this study

| **Primer pair** | **Sequence(5’-3’)** | **Cutting sites** | **Description or purpose** | |
| --- | --- | --- | --- | --- |
| Primers used for molecular cloning | | | | |
| PopA1.pHB.F/PopA1.pHB.R | CCCAAGCTTATGTCAGTCGGAAACATCC  GCTCTAGATTACATCGGCTGCGTCGAG | *Hin*dIII-*Xba*I | A 1035-bp full length *PopA1* cloned into pHB | |
| PopA3.pHB.F/PopA3.pHB.R | CCCAAGCTTATGTCGGCCAACAAGACCGG  GCTCTAGATTACATCGGCTGCGTCGAG | *Hin*dIII-*Xba*I | A 750-bp DNA fragment coding for *PopA3* cloned into pHB | |
| PopA1.pET41.F/PopA1.pET41.R | CGGAATTCATGTCAGTCGGAAACATCCAG  CCCTCGAGCATCGGCTGCGTCGAGGTG | *Eco*RI-*Xho*I | A 1035-bp coding sequence of *PopA1* cloned into pET41a(+) | |
| PopA3.pET41.F/PopA3.pET41.R | CGGAATTCATGTCGGCCAACAAGACCGG  CCAAGCTTCATCGGCTGCGTCGAGGTG | *Eco*RI-*Hin*dIII | A 750-bp coding sequence of *PopA1* cloned into pET41a(+) | |
| PopA1.BD.F/ PopA1.BD.R | CCCATATGATGTCAGTCGGAAACATCC  CGGAATTCTTACATCGGCTGCGTCGAG | *Nde*I-*Eco*RI | A 1035-bp coding sequence of full length *PopA1* cloned into pGBKT7 | |
| atpA.AD.F/atpA.AD.R | CCCATATGGAACTTTCTCCCCGAG  CGGAATTCTTAAATAAAAGCTAAAGCACT | *Nde*I-*Eco*RI | A 1530-bp coding sequence of full length *atpA* gene cloned into pGADT7 | |
| nLUC-atpA.F/nLUC-atpA.R | CGGGGTACCATGGAACTTTCTCCCCGA  GCGTCGACAATAAAAGCTAAAGCACTTTCTTTT | *Kpn*I*-Sal*I | A 1530-bp coding sequence of full length *atpA* gene cloned into pCAMBIA-NLuc | |
| cLUC-PopA.F/cLUC-PopA.R | TCGGTACCTCAGTCGGAAACATCCAGAG  AGGGTACCTTACATCGGCTGCGTCGAGGT | *Kpn*I | A 1032-bp full length *PopA1* with the deletion of translation start codon ATG cloned into pCAMBIA-CLuc | |
| atpA.RNAi.F/atpA.RNAi.R | TGGGATCCCCGAGCTGCGGAACTAA  TCTCTAGACGCCCTAGCATAGCCTTTC | *Bam*HI*-Xba*I | A 309-bp fragment *atpA* gene cloned into TRV2 | |
| PatpA.F/PatpA.R | GCGGATCCCTTTCCCACTCCCTTTGATT  TCCTGCAGCTCGGGGTGAAAGTT | *Bam*HI-*Pst*I | A 1488-bp promoter sequence of *atpA* cloned into pCAMBIA1381 | |
| PopA1.RFP.F/ PopA1.RFP.R | TGCTCGAGCAGTCGGAAACATCCAGAG TCAAGCTTTCGGCTGCGTCGAGGTGGA | *Xho*I-*Hin*dIII | A 1035-bp coding sequence of *PopA1* cloned into pGDR | |
| atpA.YFP.F/ atpA.YFP.R | TCCTCGAGAACTTTCTCCCCGAGCTG  TCAAGCTTTTAAATAAAAGCTAAAGCACT | *Xho*I-*Hin*dIII | A 1530-bp coding sequence of *atpA* cloned into pGDY | |
| atpA.GFP.F/ atpA.GFP.R | CCCTCGAGATGGAACTTTCTCCCCGA  GCGTCGACGAGGAGTGTCGCTCCTGG | *Xho*I-*Sal*I | A 1530-bp coding sequence of *atpA* cloned into pGDGm | |
| PopA1.Yn.F/ PopA1.Yn.R | TCTCTAGAATGTCAGTCGGAAACATCCA  TTGGTACCCATCGGCTGCGTCGAGGT | *Xba*I*-Kpn*I | A 1035-bp DNA fragment coding for *PopA1* cloned into 1301-YN | |
| atpA.Yc.F/ atpA.Yc.R | TCTCTAGAATGGAACTTTCTCCCCGAG  TCGGTACCTTAAATAAAAGCTAAAGCACT | *Xba*I*-Kpn*I | A 1530-bp coding sequence of *atpA* cloned into 1301-YC | |
| Primers used for qRT-PCR analysis | | | | |
| **Gene** | **Sequence(5’-3’)** | | | **Description** |
| *atpA* | GGTCTGCCGCTCAGTTGAA; AGCATCAAGGTCTGAGCCAAAT | | | 125-bp |
| *gusA* | TAGAAACCCCAACCCGTGAA; TTGCCCGGCTTTCTTGTAAC | | | 120-bp |
| *EF1a* | TGGTGTCCTCAAGCCTGGTATGGTTG; ACGCTTGAGATCCTTAACCGCAACATTCTT | | | 160-bp |
| *hin1* | CTGCAACCCATGTAGCTGTCTC; TTTGTTAGGACGAAGAACGAGCC | | | 124-bp |
| *hsr203* | TGGCTCAACGATTACGCAGAT; GACGGCAACTTGGTGGACTA | | | 84-bp |
| *PR1a* | ATGGTCAATACGGCGAAAAC; CCTAGCACATCCAACACGAA | | | 188-bp |

The 5′ end of each primer contains a restriction enzyme site for cloning into the expression plasmids
