# Supplementary material for: Ralstonia solanacearum elicitor RipX Induces Defense Reaction by Suppressing the Mitochondrial atpA Gene in Host Plant
Source: Int J Mol Sci. 2020 Mar 15;21(6):2000. doi: 10.3390/ijms21062000 (PMC7139787; doi:10.3390/ijms21062000)
Supplement: Supplementary file 1 [file ijms-21-02000-s001.zip › Figures S1-S3.docx]

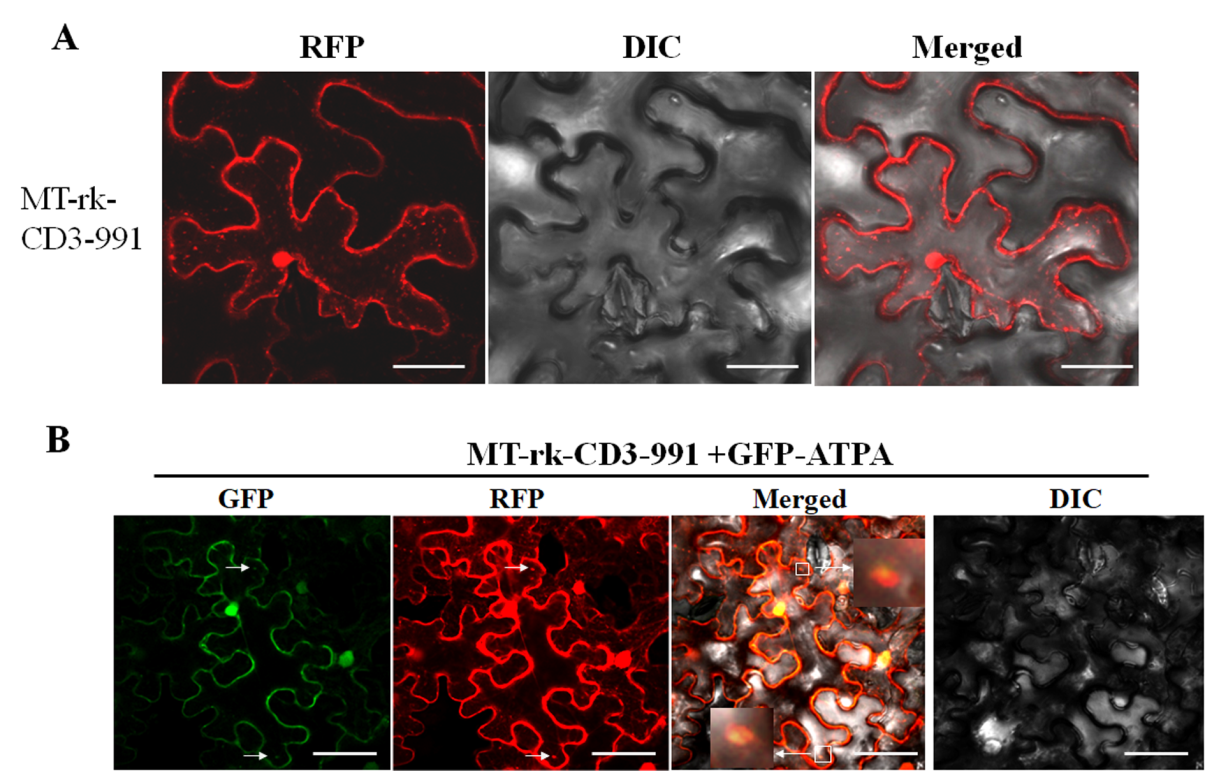


**Figure S1.** The mitochondrial targeting characteristic of ATPA. (**A**) RFP signal detected in *N. benthamiana* cells expressing the MT-rk-CD3-991 construct. (**B**) Colocalization of MT-rk-CD3-991 and GFP-ATPA in *N. benthamiana* cells. The arrows indicate the mitochondria colocalized with MT-rk-CD3-991 and GFP-ATPA. Enlarged views are indicated in the merged image. Samples were examined under a confocal microscope 2 days after agroinfiltration. All experiments were repeated three times. The scale bar represents 50 μm.


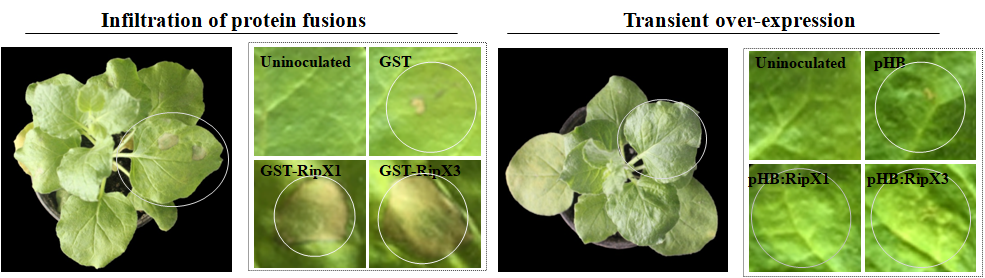


**Figure S2.** Examination of *gfp*-silenced plants in response to RipX. The responses were studied by both *Agrobacterium*-mediated transient overexpression and external infiltration of 0.05 μg/μl RipX fusion proteins.


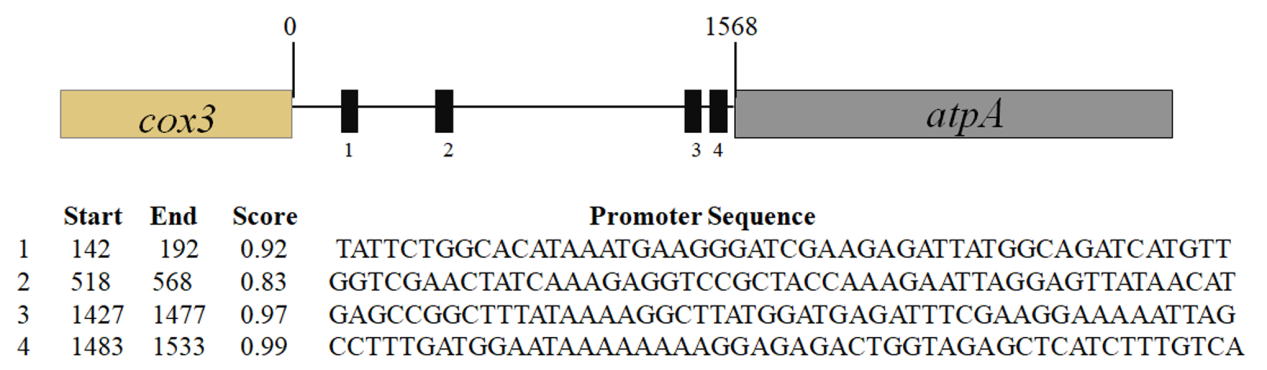


**Figure S3.** Sequence analysis of the *atpA* promoter region. The promoter elements are shown in the upper line, and the localizations are indicated in the schematic diagram.
